# Synthetic biology-inspired cell engineering in diagnosis, treatment, and drug development

**DOI:** 10.1038/s41392-023-01375-x

**Published:** 2023-03-11

**Authors:** Ninglin Zhao, Yingjie Song, Xiangqian Xie, Ziqi Zhu, Chenxi Duan, Cheng Nong, Huan Wang, Rui Bao

**Affiliations:** 1 Division of Infectious Diseases, State Key Laboratory of Biotherapy and Center of Infectious Diseases, West China Hospital, Sichuan University, Chengdu, China; 2 College of Life Science, Sichuan Normal University, Chengdu, China; 3 State Key Laboratory of Coordination Chemistry, Chemistry and Biomedicine Innovation Center of Nanjing University, Jiangsu Key Laboratory of Advanced Organic Materials, School of Chemistry and Chemical Engineering, Nanjing University, Nanjing, China

**Keywords:** Cancer, Drug delivery, Biomarkers, Genetic engineering

## Abstract

The fast-developing synthetic biology (SB) has provided many genetic tools to reprogram and engineer cells for improved performance, novel functions, and diverse applications. Such cell engineering resources can play a critical role in the research and development of novel therapeutics. However, there are certain limitations and challenges in applying genetically engineered cells in clinical practice. This literature review updates the recent advances in biomedical applications, including diagnosis, treatment, and drug development, of SB-inspired cell engineering. It describes technologies and relevant examples in a clinical and experimental setup that may significantly impact the biomedicine field. At last, this review concludes the results with future directions to optimize the performances of synthetic gene circuits to regulate the therapeutic activities of cell-based tools in specific diseases.

## Introduction

Modern science is becoming highly multidisciplinary to promote the innovation of new theories and technologies. Synthetic biology (SB) is one of the fastest-growing and most dynamic multidisciplinary fields comprising different projects, approaches, and definitions.^1–3^ SB applies engineering principles to genetically design and transform cells or living organisms by constructing biological functional components, devices, and systems, creating or reinventing biological modules to meet human needs, or even creating novel biological systems.^4^ SB-inspired cell factory engineering for industrial applications, in a narrow concept, is about using renewable biomass resources as raw materials and synthetic assembly of existing components from microbial or animal cells to produce various products.^5–7^ Engineered and standardized functional modules are the strengths of SB. Appropriate interface design makes modules detachable, improving their stability and compatibility in different environments. Whole-genome synthesis, redesign, and reconstruction allow generating of artificial biomimetic/minimal cells, while atomic or module/domain-level protein engineering offers high specificity and controllability over more complex functions. Meanwhile, innovative modification and creative assembly of signaling and communication systems can further optimize cell engineering. In addition, mathematical models for functional module simulation and prediction help streamline circuit design for the development of invaluable research tools.^8–10^

Engineered cells can have various applications, including the production of industrial or pharmaceutical compounds and increasing agricultural productivity by alleviating pathogens and environmental challenges.^11,12^ SB-inspired cell engineering can be commercialized for medical and healthcare. The main goal of medical SB is to genetically modify cells and redesign/synthesize regulatory systems to assist in disease diagnosis and treatment.^13,14^ Such cell-based sensors and therapies could achieve more specific detection and treat diseases on a much broader scale.^12,15,16^ Medical SB utilizes rationally designed therapeutic gene circuits, which can be implanted into the human body via vectors to correct the targeted defect. SB-inspired cell engineering with efficient design, specificity, and control can maximize therapeutic effects while minimizing side effects.

**Fig. 1:**
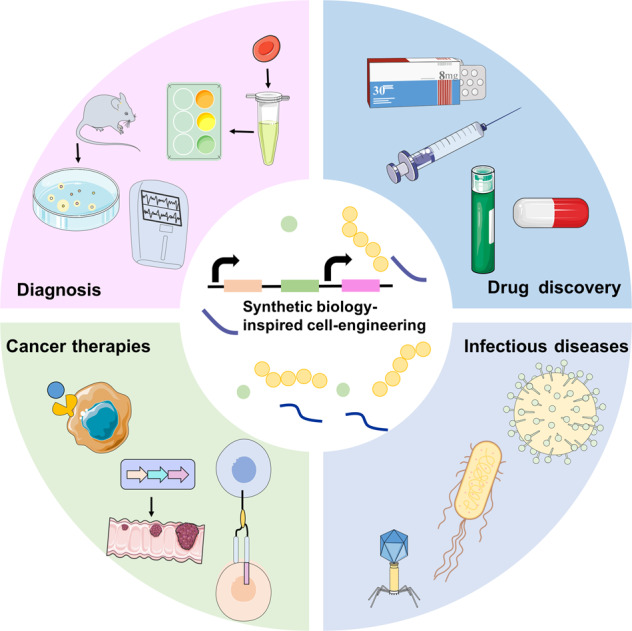
Synthetic biology-inspired cell engineering can be employed for various medical applications. Synthetic gene networks are uploaded into cells for disease diagnosis, cancer therapies, infectious diseases treatment, and drug discovery

There are several reviews on SB technologies for developing therapeutic strategies or improving clinical effectiveness; most of them covered different aspects or specific approaches of SB in medical applications (Fig. 1).^17–26^ The ongoing development of engineered cells-derived tools has attracted substantial public attention. Accordingly, this review summarizes the recent preclinical, clinical, and experimental data to discuss the benefits and limitations of SB techniques in cell engineering and biomedical research. Here, we elaborate on various SB-driven cell devices in diagnosis, treatment, and drug development. Also, we address the current and potential future challenges for SB and cell engineering in medical applications.

## Engineered cells for diagnosis

**Fig. 2:**
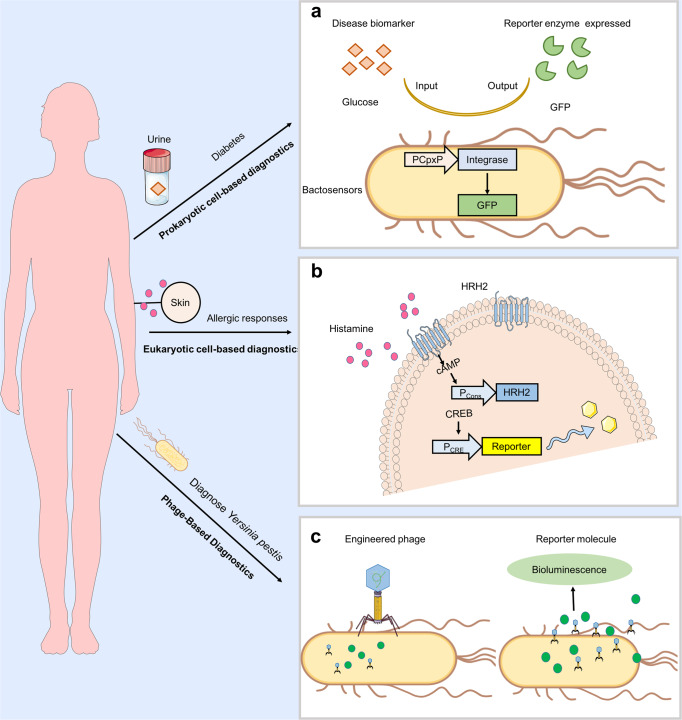
Synthetic biology platforms for diagnostics. **a** Schematic representation of engineered bacteria sense-and-respond systems adopt disease biomarkers (orange quadrilateral) to sense disease, in turn, produce a diagnostic readout such as β-lactamase and green fluorescent protein (GFP) (green Pacman). **b** Applications of mammalian synthetic biology in diagnostics. Monitoring of allergic immune by engineered mammalian cell encoding the histamine sense-and-response device. The constitutively expressed histamine-responsive receptor HRH2 sense histamine and triggers an endogenous signaling cascade leading to expression of a reporter protein. **c** Phage-based diagnostics rely on engineered phage can specific recognition of target bacterial species. Once the engineered phage bound to the target cells, the genome is injected into it then the phage replicate and reporter gene expressed

Accurate disease characterization and monitoring are critical for therapeutic planning and epidemiological records. However, most traditional diagnostic procedures involving pathological examination of biopsies cannot precisely detect the disease state in real-time and are time-consuming. Thus, there is an urgent need to enhance current medical diagnostics capabilities. SB-inspired cell engineering and programmable biosensors have focused on high-throughput biomolecular/pathogens identification and probing, which can improve clinical diagnosis and disease management protocols (Fig. 2 and Table 1).^25,27,28^ Here, we describe the emerging advances of SB-inspired cell engineering approaches in diagnostics that may speed up data acquisition with improved sensitivity, specificity, real-time quantification, and accuracy at a substantially lower cost.

**Table 1:** Advances in engineering cells for disease diagnosis

Disease	Biomarker	Reporter	Diagnostics feature
Zinc deficiency	Zinc	Pigments	Zinc-responsive transcription factors Zur and ZntR are modules to respond extracellular concentrations of zinc^37^
Diabetes	Glucose	Fluorescence	Glucose-responsive integrase expression cassette (PCpxP–integrase–pA) that expresses the green fluorescent protein (GFP) for glycosuria testing^38^
*Vibrio cholera* infection	Cholera autoinducer 1 (CAI-1)	Colorimetric enzymatic reaction	CqsS-NisK quorum sensing block for cholera autoinducer 1 (CAI-1) detection^44^
*Staphylococcus aureus* infection	Autoinducer peptide-I (AIP-I)	Yellow pigment p-nitrophenol	Agr quorum sensing (agrQS) from *Staphylococcus aureus* to detect autoinducer peptide-I (AIP-I)^45^
Gut inflammation	Thiosulfate/tetrathionate	Colorimetric enzymatic reaction	Two-component signaling system ThsSR/TtrSR is engineered to detect thiosulfate/tetrathionate^49,50^
Gut inflammation	Nitrate	Fluorescence	NarX-NarL two-component system in *E. coli* responds to nitrate^51^
Liver tumors	Liver metastases	Bioluminescent	PROP-Z (programmable probiotics with lacZ) platform can report liver metastasis^52^
Intestinal bleeding	Heme	Fluorescence	Probiotic biosensor based on the sensitivity to heme diagnosis of gastrointestinal bleeding in pigs^53^
Allergic responses	Histamine	Citrine	Engineered HEK293 cells can sensing the extracellular histamine levels and triggering cAMP-induced SEAP (secreted alkaline phosphatase) expression^65^
Hypercalcaemia	Calcium	Melanin	Engineered HEK293 cells monitor calcium levels and produce melanin visible through the skin^66^
*Yersinia pestis*	Phage ΦA1122 with luxAB	Bioluminescence	Engineered a diagnostic phage for *Yersinia pestis* detection by bioluminescence reporter^81^
*Mycobacterium tuberculosis*	Fluoromycobacteriophages	Bioluminescence	Microscopy-based method utilizing the reporter mycobacteriophage mCherrybombϕ for detection of *M. tuberculosis*^82^
*Listeria monocytogenes*	Nanoluciferase (NLuc) -based phage	Bioluminescence	Engineered nanoluciferase (NLuc)-based phage reporters to identify *Listeria monocyte*^83^

### Prokaryotic cell-based diagnostics

In the past several decades, bacteria-based biosensors have been extensively used in the food, environment, and medical industries.^29,30^ Bacterial biosensors can rapidly adapt to test environments to sense biological substances both in vitro and in vivo. Bacterial sensors are preferred for selectivity, sensitivity, automation, miniaturization, and accuracy.^31^ Engineered bacterial sensors have been successfully used for the analysis of environmental pollutants.^32,33^ Many bacterial sensors with specific and sensitive gene circuits for the identification of biological signals can be used to detect widespread clinically relevant biomarkers in vitro, such as in serum and urine.^34^ In particular, certain symbiotic bacteria that colonize specific tissues and organs are believed to be feasible candidates for cell engineering to serve as in vivo tools for real-time diagnosis.^28,35,36^

Most bacterial-based diagnostic biosensors produce an easily measurable output in the form of a pigment or fluorescent protein upon identification of a target signal.^37,38^ For instance, Zur and ZntR zinc-responsive transcription factors (TFs) modules respond to extracellular concentrations of zinc and produce visible pigments; *Escherichia coli* cells integrated with this circuit can sense serum zinc levels to identify zinc deficiency.^37^ Such a bacterial sensor impacts multiple metabolite production pathways through a single TF, reduces diagnostic time, and explains results through visual inspection. Similarly, the glucose-responsive integrase expression cassette (PCpxP–integrase-pA), which expresses the green fluorescent protein (GFP), is an ideal reporter to test glycosuria; hydrogel beads immobilized bacteria containing this module can evaluate urine glucose levels in diabetic patients with high sensitivity and specificity.^38^

Quorum sensing (QS) systems that determine population behavior are widely distributed among bacteria and have a long evolutionary history.^39^ QS signaling molecule profiling from specific pathogens are proper biomarkers for bacterial diagnosis; acyl-homoserine lactone (AHL), 2-heptyl-4-quinolone (HHQ), and acyl-homoserine lactones (acyl HSLs) have been used to identify *P. aeruginosa* infection.^40–42^ Importantly, QS signals can be directly monitored by genetically engineered non-pathogenic bacteria.^43^ Mao et al. reprogrammed the probiotic *Lactococcus lactis* by introducing a chimeric CqsS-NisK quorum sensing block for the detection of cholera autoinducer 1 (CAI-1) from *Vibrio cholerae*.^44^ This system can easily detect *V. cholerae* in fecal samples and intestinal environments. Another research group generated a probiotic *Lactobacillus reuteri* strain integrated with an agr quorum sensing (agrQS) biosensor from *Staphylococcus aureus*, which could identify quorum sensing molecule autoinducer peptide-I (AIP-I) from common pathogenic *Staphylococcus sp* with high sensitivity in the nanomolar range.^45^ Engineered probiotic bacteria integrated with a rational antimicrobial module can be novel therapeutic “robots” to track and eliminate pathogenic bacteria.

Two-component system (TCS), including a membrane-bound histidine kinase for signal sensing/transduction and a cognate intracellular response regulator to activate downstream gene expression, is a key mechanism of how bacteria perceive and respond to the environment.^46^ The well-defined TCSs have been used for diagnostic signal analysis.^47^ Holowko et al. integrated three *V. cholerae*-derived TCS elements CqsS, LuxU, and LuxO with a dCAS9-based GFP-reporter system into *E. coli* to construct a biosensor with a high sensitivity for QS-ligand cholera CAI-1 indicating the presence and proliferation of *V. cholera*.^48^ Previous studies engineered thiosulfate/tetrathionate-responsive *E.coli* strain by combining TCS sensors ThsSR/TtrSR with pre-installed reporter system or phage lambda cI/Cro-based memory system as potential therapeutics and diagnostics for inflammation^49,50^ Recently, a new TCS-based whole-cell biosensor was designed by adopting *E. coli* NarX-NarL to sense nitrate levels, a biomarker of gut inflammation.^51^ This highly specific nitrate-responsive genetic circuit allows non-invasive diagnosis of inflammation-related diseases.

In addition to in vitro diagnosis, engineered bacteria systems can also be exploited for the diagnosis of complex internal environments. Biomolecular monitoring of the gastrointestinal environment is often limited by access and complexity of the environment. Danino et al. developed a PROP-Z (programmable probiotics with lacZ) platform using a Nissle 1917 (EcN) derived bacterium with a series of expression cassettes. This orally administered probiotic can produce detectable signals in urine reliably indicating hepatic tumors.^52^ Another exquisitely engineered micro-bio-electronic device (IMBED) was developed by Mimee et al. They efficiently combined a bacterial-electronic system with SB circuits, electronic sensor, and wireless transmission platform to detect excess heme in blood samples offering rapid in situ diagnosis of gastrointestinal bleeding in pigs.^53^

### Eukaryotic cell-based diagnostics

Microbial biosensors offer inexpensive and feasible disease detection strategies. Similar to engineered bacteria, eukaryotic cell-based biosensors have also been developed for real-time clinical applications.

The number of yeast/fungi-based diagnostic biosensors is growing. Instead of using optical signals such as fluorescence, luminescence, and colorimetry, many yeast-based biosensors utilize electrochemical elements (amperometry, potentiometry, conductometry, voltammetry) and growth indicating modules (HIS3, TRP1, LEU2).^54–56^ One of the advantages of eukaryotic cell biosensors is to incorporate G-protein-coupled receptors (GPCRs) which greatly expands their functional repertoire.^57–59^ Diagnostic yeast biosensors have often been used to detect the metabolic rate of oxygen, glucose, lactate, and ethanol through enzyme-catalyzed oxidation-reduction reactions; the generated electrical energy is measured by an implemented electrode which rapidly generates signals with high sensitivity and specificity. A d-lactate-selective biosensor was developed from the debris of the recombinant thermotolerant methylotrophic yeast *H. polymorpha*. This biosensor overproduces d-lactate: cytochrome c-oxidoreductase (DLDH) and has high selectivity and effective stability for d-lactate due to the deletion of cytochrome c-oxidoreductase (CYB2, responsible for l-lactate synthesis) in the C-105 strain genotype (gcr1 catX).^60^ The same research group further suggested that enriching the flavocytochrome b2 (FC b2) module incorporated *H. polymorpha* cells by enzyme-bound nanoparticles can improve amperometric current responses.^61^

Combining new ideas and technologies based on existing yeast-based biosensors stimulates more innovations. Holly V. Goodson’s research group designed an *S. cerevisiae*-based olfactory reporter by assembling the endogenous galactose-response system and the gene encoding acetyltransferase I (ATF1, isoamyl alcohol-converting enzyme). This system produces enough amount of isoamyl acetate (banana flavor) in response to galactose and is suitable for low-cost non-quantitative/semi-quantitative diagnosis.^62^ Furthermore, biological paper analytical devices (bioPADs) were created by embedding this bioengineered fluorescent yeast biosensor into a portable paper substrate. This simple but sensitive test strip can detect doxycycline in complex matrices such as in human urine and raw bovine serum.^63^ It is foreseeable that sustainable developments in fungal genome engineering would take yeast biosensors toward precise, accurate, and cost-effective editing.

Based on the availability and compatibility of endogenous signal-transduction biomarkers, engineered mammalian cells are more suitable for certain diagnostics applications over bacterial and yeast biosensors.^64^ With the recent technological advances, a few mammalian cell-based diagnostics have been developed. For example, HEK293 cell-based biosensor with a synthetic histamine-responsive signal module (histamine receptor H2, HRH2) can detect allergies from blood samples of high-risk allergic patients by sensing the extracellular histamine levels and triggering the expression of cAMP-induced SEAP (secreted alkaline phosphatase).^65^ Such engineered mammalian cell systems can be used in high-throughput diagnostic applications such as testing novel drugs for allergic reactions. Many diseases are preceded by a period of asymptomatic pathogenesis and thus require reliable indirect molecular diagnosis methods. Accordingly, Martin Fussenegger’s research group designed a novel HEK293 cell for in vivo diagnostics by employing a calcium-sensing receptor constructed with a synthetic signaling pathway. In response to the increased calcium level in hypercalcemia, this system generates black pigment melanin on the skin via transgenic tyrosinase-mediated enzymatic reaction and thus is ideal for naked-eye detection.^66^

Except for epithelial cell lines, other mammalian cells including macrophages, neurons cell, mast cells, and B lymphocytes can also be exploited for biosensor applications. Based on the pattern of signal detection and test objective, a specific assay combined with proper cell lines can be used to assess pathogens, toxins, metabolic activity, or even cell proliferation.^67^ Monkey kidney cells (Vero cells) are sensitive to verotoxin Stx (Shiga-like toxins) due to their overproduced Stx receptor GB3 (globotriaosylceramide).^68^ Bioengineered Vero cells constructed in collagen matrix demonstrated better cell maturation and improved sensitivity to Stx and thus could detect Shiga-like toxin-producing pathogenic bacteria such as *Salmonella, Listeria, Citrobacter, Serratia,* and *Hafnia*.^69^ IgE-mediated vasoactive amines including histamine and serotonin degranulation are characteristics of basophil or mast cells, which make them a good choice for cell-based biosensor development.^70^ Jiang et al. reported an electrochemical sensor based on rat basophilic leukemia (RBL-2H3) cells exploited on folding paper-based equipment for casein detection.^71^ Certainly, engineered mammalian cell-based devices for therapeutic and diagnostic applications are a growing area of interest. The diverse cell behaviors in response to different compounds such as antibiotics, antibodies, and chemokines make mammalian cells attractive model for building diagnostic biosensors. Meanwhile, the complexity and packaging of eukaryotic genome have also to be considered when using them for such applications.

### Phage-based diagnostics

Although cell-based biosensor diagnostics can fulfill most needs of clinical applications, the rational design of engineered phages promises some other advantages. Phages are viruses that infect bacteria by adhering to specific host cell surface receptors. A simple model for reporter phage design is that the presence of the correct epitope induces the transfer of the phage genome into bacteria, triggering bioluminescence from luciferase in the target bacteria.^72,73^ Bioluminescent reporter-based phages have been applied for the diagnosis of pathogens such as *Bacillus anthracis*,^74,75^ *Vibrio parahaemolyticus*,^76^ *Salmonella typhimurium*,^77^ *pathogenic Escherichia coli*,^78,79^ and *Staphylococcus aureus*.^80^ For example, an engineered *Yersinia pestis* phage was used for the detection of an etiologic agent in bubonic plague.^81^ A microscopy-based method in association with the fluoromycobacteriophage mCherrybombϕ was used to diagnose *M. tuberculosis*.^82^ Recently, Meile et al. engineered a set of nanoluciferase (NLuc)-based phage reporters to identify and differentiate *Listeria monocytogenes* in potentially contaminated milk, cold food, and other eatable samples.^83^ These NLuc-based reporter phages enabled rapid and ultrasensitive detection of one CFU of *L. monocytogenes* in 25 g of artificially contaminated natural food in less than 24 h.

Phages can also be exploited as bioelectrode elements by immobilizing them to an electrode surface or utilizing lytic phages to infect target bacteria to release bacterial cell contents such as adenosine triphosphate (ATP) for electrochemical detection.^84^ Niyomdecha et al. used an M13 bacteriophage-modified electrode for the detection of *Salmonella spp.:* this biosensor binds to target bacteria through the amino acid groups on the filamentous phage.^85^ Farooq et al. mobilized *S. aureus*-specific lytic phages in surface-modified bacterial cellulose (BC) matrix conducted by carboxylated multi-walled carbon nanotube (c-MWCNTs). The functionalized electrode could detect *S. aureus* through impedance measurements.^86^ Lately, gold electrodes were used as a transducer with chemically modified M13 phages to monitor caspase-3 activity in apoptotic HeLa cells with nanomole range sensitivity.^87^ Such bacterial cell and phage-based biosensors can effectively sense disease-associated biomarkers, paving the way for next-generation of medical diagnostics.

## Engineered cell-based therapeutics for cancer

More than 19 million new cases of cancer are diagnosed every year, making it one of the most devastating diseases worldwide.^88–90^ Traditional cancer treatments such as surgical intervention, radiation, and chemotherapeutic drugs have several limitations, including high cytotoxicity and low selectivity.^91–94^ SB approaches for cancer treatment are the most vivid and promising trends that enable precise timing, location, and dosing of the drug. Accordingly, engineered mammalian and bacterial cells have been increasingly recognized as promising anticancer devices.^95–97^

### Bacteria-derived tools for cancer treatment

**Fig. 3:**
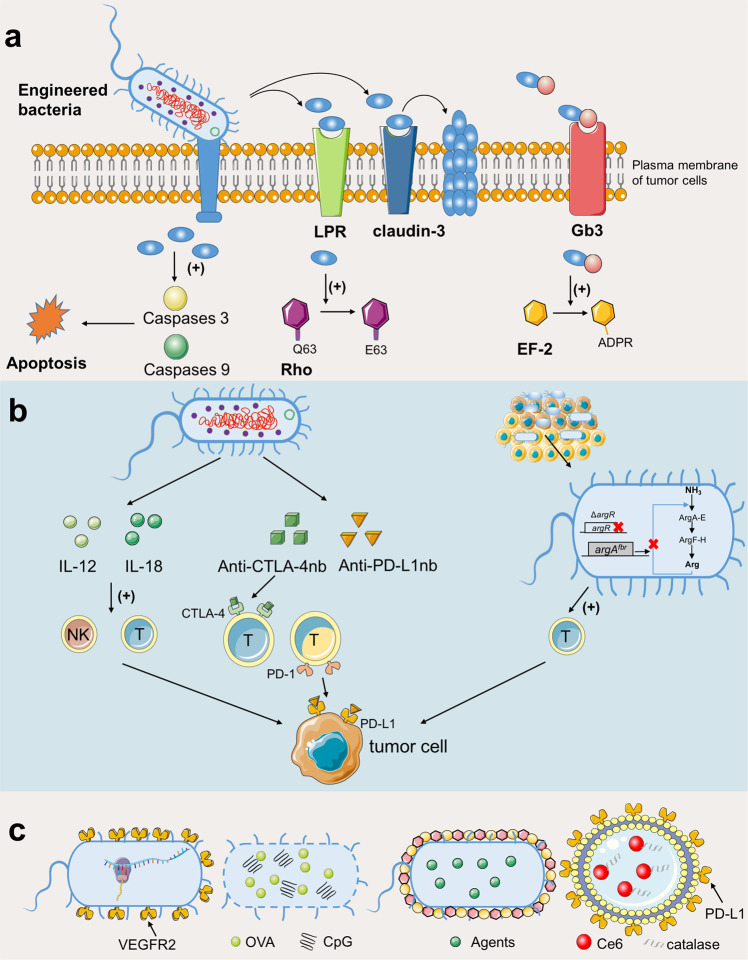
Engineered bacteria for cancer therapy. Current strategies can be divided into three types: **a** expression of toxic proteins to directly kill cancer cells, **b** immunotherapeutics, and **c** therapeutic drug delivery vehicles. **a** Bacteria could utilize secretion systems such as T3SS and T6SS to directly inject bacterial effector proteins into eukaryotic host cells, or secret effectors targeting receptors on cell surface. LRP laminin receptor precursor protein, EF-2 elongation factor 2. **b** Engineered bacteria could express cytokines or other chemical molecules to induce proper immune cells. IL-12 Interleukin 12, CTLA-4 cytotoxic T-lymphocyte-associated antigen-4, PD-1 programmed death-1, PD-L1 programmed cell death protein-ligand 1, nb nanobody. **c** Bacteria-based vaccine or drug delivery systems. VEGFR2 vascular endothelial growth factor 2, OVA ovalbumin

Since some bacteria can colonize in a hypoxia intratumoral environment, human cancer can now be treated by SB-engineered bacteria.^98–101^ Certain bacterial species, including *Streptococcus*, *Caulobacter*, *Escherichia*, *Bifidobacterium*, and *Salmonella*, can automatically sense and accumulate in tumor tissues after external administration (injection or oral) to produce cytotoxic toxins or release delivery compounds.^102^ One of the major advantages of bacterial therapies for cancer is that they can be easily manipulated for the effective delivery of anticancer agents.^103^ Based on the reported studies, bacterial therapies for cancer majorly involves three types of mechanisms: expression of toxic proteins to directly kill cancer cells, immunotherapeutics that stimulate the immune system to kill cancer cells or the transfer of therapeutic drug delivery vehicles (Fig. 3 and Table 2).^102,104,105^

**Table 2:** Bacteria-based therapies against cancer in clinical trials

Type	Biological/tradename	Sponsor	NCT	Year	Phase	Status	Cancer type
*Salmonella Typhimurium*	VNP20009	National Cancer Institute	NCT00004988	2008	I	Completed	Cancer neoplasm, neoplasm, metastasis^297^
	VNP20009	National Cancer Institute	NCT00006254	2013	I	Completed	Unspecified adult solid tumor^298^
	Salmonella-IL-2	Masonic Cancer Center, University of Minnesota	NCT01099631	2020	I	Completed	Solid tumor cancer^299^
	VXM01/Avelumab	Vaximm GmbH	NCT03750071	2021	I/II	Recruiting	Recurrent glioblastoma
	SGN1	Guangzhou Sinogen Pharmaceutical Co., Ltd.	NCT05103345	2021	I	Not yet recruiting	Advanced solid tumor
	Salmonella-IL-2	Salspera LLC	NCT04589234	2022	II	Recruiting	Metastatic pancreatic cancer
	VNP20009-M	Guangzhou Sinogen Pharmaceutical Co., Ltd.	NCT05038150	2022	I	Not yet recruiting	Advanced solid tumor
*Escherichia coli*	SYNB1891/Atezolizumab	Synlogic	NCT04167137	2021	I	Recruiting	Metastatic solid neoplasm, lymphoma^300^
	VAX014	Vaxiion Therapeutics	NCT03854721	2020	I	Recruiting	Urothelial carcinoma of the urinary bladder^301^
*Clostridium novyi*-NT	*Clostridium novyi*-NT spores	BioMed Valley Discoveries, Inc.	NCT01118819	2016	I	Terminated	Solid tumor malignancies
	*Clostridium novyi*-NT spores	BioMed Valley Discoveries, Inc.	NCT01924689	2019	I	Completed	Solid tumor malignancies^302^
*Bifidobacterium longum*	bacTRL-IL-12	Iqvia Pty Ltd.	NCT04025307	2021	I	Terminated	Solid tumors
	APS001F/Flucytosine (5-FC)/10% maltose	Anaeropharma Science, Inc.	NCT01562626	2021	I/II	Suspended	Tumors, neoplasms, cancer
*Bifidobacterium animalis* *lactis*	EDP1503/Pembrolizumab	Evelo Biosciences, Inc.	NCT03775850	2021	I/II	Completed	Colorectal cancer metastatic, triple-negative breast cancer, non-small cell lung cancer^303^ Bladder cancer, gastroesophageal cancer, renal cell carcinoma, MSI-H
*Listeria monocytogenes*	ADU-623	Providence Health & Services	NCT01967758	2018	I	Completed	Astrocytic tumors^304^
	CRS-207 with Epacadostat	Aduro Biotech, Inc.	NCT02575807	2019	I/II	Terminated	Platinum-resistant ovarian, fallopian, or peritoneal cancer
	ADXS31-142+ Pembrolizumab	Advaxis, Inc.	NCT02325557	2020	I/II	Unknown	Prostate cancer
	CRS-207 in combination with chemotherapy	Aduro Biotech, Inc.	NCT01675765	2020	I	Completed	Malignant pleural mesothelioma^305^
	ADXS11-001	Andrew Sikora	NCT02002182	2021	II	Active, not recruiting	HPV-positive oropharyngeal cancer^306^
	CRS-207	Sidney Kimmel Comprehensive Cancer Center at Johns Hopkins	NCT03190265	2022	II	Active, not recruiting	Pancreatic cancer^307^
	ADXS-503 with or without pembro	Advaxis, Inc.	NCT03847519	2022	I/II	Recruiting	Metastatic non-small cell lung cancer
Multi-species/strain bacteria	Dietary supplement: probiotic	Mayo Clinic	NCT03358511	2020	Not applicable	Completed	Breast cancer^308^
	MRx0518	4D pharma plc	NCT03637803	2021	I/II	Recruiting	Oncology, solid tumor, non-small cell lung cancer, renal cell carcinoma, melanoma, bladder cancer
	VE800 with Nivolumab	Vedanta Biosciences, Inc.	NCT04208958	2021	I/II	Active, not recruiting	Metastatic cancer

Natural bacterial toxins are emerging as potential anticancer agents to treat established solid tumors.^104^ Bacterial extract from *Streptococcus pyogenes* and *Serratia marcescens* was shown effective against sarcomas, melanomas, and myelomas, and named “Coley’s toxins” based on the name of the researcher.^106,107^ Since then, more bacterial toxins have been identified and tested for anticancer therapy.^102,104^ Cytolethal distending toxins from *Salmonella typhi* and the cycle-inhibiting factor (Cif) from enterohemorrhagic *E. coli* can inhibit cancer cell growth by blocking mitosis.^108,109^ The bifunctional *Pseudomonas aeruginosa* exotoxin T (ExoT) consists of a GTPase-activating protein (GAP) and an ADP-ribosyltransferase encoded from the N- and C-terminus, respectively.^110,111^ The GAP protein guides the mitochondrial dysfunction in the host cell through activating host caspases 3 and 9, while the ADP-ribosyltransferase domain upon activating the CT10 regulator of the kinase (Crk) leads to atypical anoikis apoptosis.^112,113^ Clostridium perfringens enterotoxin (CPE) can treat colorectal cancers by binding to claudin-3 on the tumor cell surface forming a multi-protein membrane pore complex, altering cellular osmotic equilibrium that leads to cell lysis.^114,115^ To improve the specific recognition of bacterial toxins for cancer cells, they can be reprogrammed either to conjugate with tumor surface antigens or ligands that selectively bind to the target cell receptor.^104,116^ For instance, *P. aeruginosa* exotoxin A (PE) was fused to anti-CD25 to form a novel recombinant toxin.^117^ Native diphtheria toxin (DT) was linked with the B-subunit of Shiga-like toxin (STXB) to improve its affinity for breast cancer cells and showed improved cytotoxicity against Gb3-expressing cancer cells in a dose-dependent manner.^118^ Besides, bacterial spores can also be exploited as anticancer agents as they can specifically germinate inside the necrotic/hypoxic tissues such as a solid tumor.^119,120^ *Clostridium histolyticum* spores showed considerable oncolysis and tumor regression effects in murine models.^121^ However, the original bacterial spores might not eliminate cancer cells and would require genetic modifications or combinational use with other anticancer strategies such as radiotherapy and chemotherapy.

Expression of exogenous cytotoxic proteins may trigger the host immune response raising safety concerns while engineered bacteria to induce specific cytokines can be an alternative anticancer strategy.^102^ Selective expression of cytokines can induce proper immune cells to clear tumors via multiple mechanisms with fewer side effects.^122^ Interleukin-2 (IL-2) and IL-18 are the most crucial components of cancer-related inflammation and participate in the immune regulation network. They activate the antitumor cytotoxicity of T cells and natural killer cells.^123,124^ Engineered *Salmonella* producing IL-2 and IL-18 was shown to inhibit tumor growth in animal experiments.^125,126^ Targeting programmed cell death protein-ligand 1 (PD-L1) and cytotoxic T-lymphocyte-associated protein-4 (CTLA-4) by immune-checkpoint inhibitors is a revolutionary cancer immunotherapy strategy but has side effects such as fatigue, skin rashes, and endocrine disorders.^127,128^ Increasing l-arginine levels in tumor tissue can activate the response of immune-checkpoint inhibitors. Engineered *E. coli* Nissle 1917 strain after accumulation in the tumor tissue converted ammonia to l-arginine recruiting tumor-infiltrating T cells showing a remarkable antitumor effect.^129^ Gurbatri et al. engineered a probiotic bacteria system to tightly control the release of nanobodies, which targeted PD-L1 and CTLA-4 and activated T cells.^130^ In another case, engineered *Salmonella* (△ppGpp) was utilized to secret the development endothelial locus 1 (Del-1) protein promoting the recruitment of M1 macrophage to tumors.^131^

The deoxygenated inflammatory microenvironment of a tumor is distinct from most other tissues and therefore allows selective accumulation of obligate anaerobes in the tumor for the delivery of bacteria-based vaccine or drug.^102,105^ Hu et al. used self-assembled synthetic nanoparticles coated attenuated bacteria to develop a novel oral DNA vaccine to express vascular VEGFR2 and specific antigens.^132^ This engineered bacteria could activate T cells and induce the production of cytokine for efficacious cancer immunotherapy. In 2017, researchers exploited demi-bacteria (DB) from *Bacillus* for the efficient accommodation of antigen and cytidine-phosphate-guanosine (CpG) adjuvants, inducing synergistic cellular and humoral immune responses in vivo.^133^ In combination with traditional chemotherapy and/or phototherapy, SB approaches can significantly promote the anticancer therapeutic outcome. For instance, attenuated *Salmonella* coated with degradable polydopamine was used for the delivery of photothermal agents to tumors.^134^ To efficaciously transport the chemotherapeutic drugs to the intestinal environment, *Bacillus coagulans* spores were modified with deoxycholic acid (DA) and then load them with drugs serving as autonomous nanoparticle (NP) producers.^135^ In addition, the bacteria-derived outer membrane vesicles (OMVs) have been explored as drug carriers in vaccine developments and cancer therapies.^136,137^ Based on oxygenated PDT and immunotherapy, Zhang et al. combined Chlorin e6 (Ce6) with negatively charged catalase (CAT) to form a molecular scaffold (CAT-Ce6) and transported it using aPDL1 modified (OMV-aPDL1) *Salmonella* OMVs to inhibit tumor growth.^138^

### CAR-T-based cellular immunotherapy

**Fig. 4:**
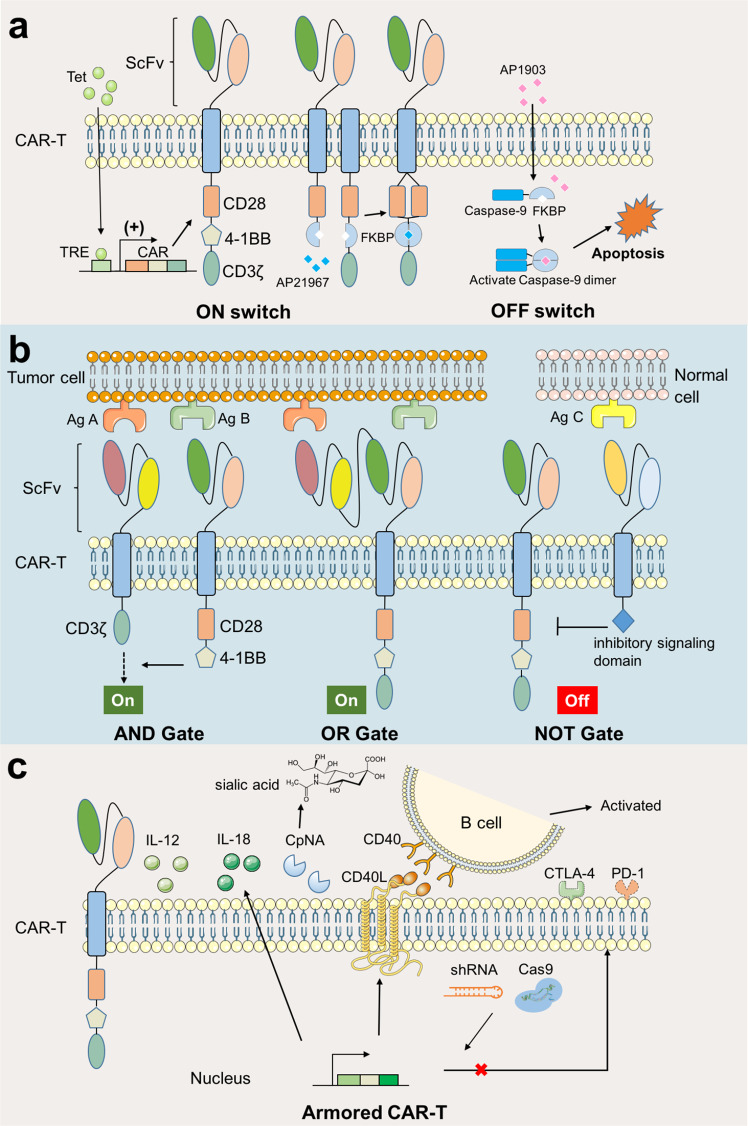
Synthetic Biology in CAR-T Cell Engineering for cancer therapy. Current strategies can be divided into three types: **a** Switchable CAR, **b** Logic gates, and **c** Armored CAR. **a** Switchable CAR includes ON switches and OFF switches. In ON switches, exogenous molecules could activate the expression of CAR elements or the recombination for activate state. In OFF switches, the exogenous molecules induce the suicide genes such as iCasp9 for controlled elimination. Tet tetracycline, TRE tetracycline response element, FKBP a rapamycin-binding protein, AP21967 rapamycin analog, AP1903 dimer inducers. **b** Logic gates includes AND Gate, NOT Gate, and OR Gate. The activation of CAR depends on the number and type of antigens from tumor cells. Ag antigen. **c** As for Armored CAR-T, CAR-T cells are engineered to expression extracellular proteins including IL-12, IL-18, other enzymes or transmembrane ligands or receptors to enhance immune response. Intracellularly engineered CAR-T cell is another powerful strategy, such as inhibiting the transcription of immune-checkpoint molecules by shRNA or CRISPR-Cas9. IL-12 Interleukin 12, CpNA *C. perfringens* neuraminidase, CD40 cluster of differentiation 40, CD40L CD40 ligand, shRNA short-hairpin RNAs, CTLA-4 cytotoxic T-lymphocyte-associated antigen-4, PD-1 programmed death-1

Engineered cellular immunotherapy has great potential for advanced hematological malignancies and other solid tumors.^139^ Compared with traditional treatment and engineered bacterial and cell-based therapies have two major advantages: (1) a large number of basic research, clinical trials, and SB can make rapid development in this field, and (2) it offers long-term precise regulation and coordination of immune response in vivo. Current cancer cell therapy includes chimeric antigen receptor (CAR)-T cell, T-cell receptor (TCR) T cell, CAR-natural killer (NK)/NKT cells, tumor-infiltrating lymphocyte (TIL), choline transporter‐like proteins, etc.^140^ Among them, CAT-T-cell therapy is one of the most innovative gene therapies with the greatest breakthrough in the field.^141,142^ At present, five cell therapies have been approved by the Food and Drug Administration (FDA) in July 2021 after clinical trials and all of them are CAR-T-cell therapies (Table 3).^140^ Claudin18.2-specific CAR-T cells (CT041) showed good efficacy against digestive tract solid tumors with 48.6% objective remission rate and 73.0% disease control rate exceeding the best result of CAR-T-cell therapy for hematoma treatment (remission rate <30%).^143^ In the last decade, CAR has been developed to the fourth generation, the present CAR consists of a single-chain variable fragment (scFv), a flexible hinge, a transmembrane domain (CD8a), one or more co-stimulatory domains, and an intracellular CD3ζ domain from the T-cell receptor.^139^ Here, we discuss the main SB strategies for antitumor CAR engineering, including switchable control, logic gates, and armored CARs, and then discuss the future possible development of cell therapies from a SB perspective (Fig. 4).

**Table 3:** Currently CAR-T products approved by FDA for cancer therapy

Product name	Target	Sponsor	First time offered to market	Approved countries	Vector	Cancer type
Kymriah (tisagenlecleucel)	CD19	Novartis	September 30, 2017	USA (2017), EU (2018), Canada (2019), Japan (2019)	Lentivirus	B-cell acute lymphoblastic leukemia^309^
Yescarta (axicabtagene ciloleucel)	CD19	Kite Pharma, Inc./Gilead Sciences lnc.	October 18, 2017	USA (2017), EU (2018), Canada (2019)	Retrovirus	Relapsed/refractory diffuse large B-cell lymphoma, relapsed/refractory primary mediastinal B-cell lymphoma, relapsed/refractory transformed follicular lymphoma, relapsed/refractory high-grade B-cell lymphoma^310^
Tecartus (Brexucabtagene autoleucel)	CD19	Kite Pharma, Inc./Gilead Sciences lnc.	October 28, 2020	USA (2020), EU (2020)	Retrovirus	Relapsed/refractory mantle cell lymphoma^311^
Breyanzi (lisocabtagene ciloleucel)	CD19	Juno Therapeutics, Inc/BMS	February 5, 2021	USA (2021)	Lentivirus	Relapsed/refractory diffuse large B-cell lymphoma, relapsed/refractory primary mediastinal B-cell lymphoma, relapsed/refractory transformed follicular lymphoma, relapsed/refractory high-grade B-cell lymphoma^312^
Abecma (idecabtagene vicleucel)	BCMA	Celgene Corporation/BMS	March 26, 2021	USA (2021)	Lentivirus	Multiple myeloma^313^
Carvykti (Ciltacabtagene autoleucel)	BCMA	Legendbiotech, Johnson & Johnson	February 28, 2022	USA (2022)	Lentivirus	Recurrent or refractory multiple myeloma^314^

Engineered switches are powerful control systems that minimize the life-threatening toxicities and other unfavorable side effects of CAR-T cells and have two types, named ON and OFF switches.^26,144^ A good example is the tetracycline regulatory platform, in which, exogenous tetracycline or doxycycline triggers the time- and dose-dependent expression of CAR components. The co-stimulatory domain could also be separated from the CAR structure and fused with small molecules binding domain for specific recognition and recombination. In addition, the antigen binding region can be engineered to recognize multiple tumor antigens, such as leucine zippers, unique epitopes, or chemical tags.^145^ In contrast to an ON switch, an OFF switch senses inhibitory signals to stop CAR T-cell activation. The drug-inducible caspase-9 (iCasp9) kill switch is one of the most successful designs, which has been used in several clinical trials for CAR T-cell therapies.^146^ The iCasp9 switch consists of a domain encoding chemically inducible dimerization system derived from FKBP12 (a rapamycin-binding protein) and split caspase-9 protein; the small-molecule AP1903 can induce the dimerization of FKBP12-caspase-9 causing apoptosis. CAR-T cells carrying the iCasp9 switch have shown effective curative effects in several clinical trials.^147,148^ There are other non-caspase strategies, such as combining MyD88 and CD40, based on the FKBP12/AP1903 dimerization system, that can overcome the limitation of elimination from the body.^149^

Considering the sophisticated tumor microenvironment and nonspecific antigens on tumor cells, more precise treatments are necessary to balance safety and efficacy. Improved genetic logic circuits, including AND Gate, NOT Gate, and OR Gate, were designed to solve these problems.^144^ All of the antigens present in AND Gate are needed for CAR-T-cell activation. Another common strategy is to separate and fuse the activation signal (CD3ζ) and the co-stimulatory domain to two different antigen-recognizing receptors, such as SynNotch system,^150^ bispecific tandem CAR (TanCAR)^151^ and masked CAR (mCAR),^152^ however, this also has the disadvantage of slow activation kinetics. Following the principle “A and not B”, NOT Gate can restrict off-target attacks of T cells reducing unintended immune attacks; however, this has not been validated in clinical trials so far. Alternatively, the purpose of OR Gate is to overcome antigen heterogeneity and antigen escape, thus multiple-targeting CAR-T therapy is developed by fusing different CAR-T cells or generating specific T cells with different CAR suits. The other two alternative strategies are combining different antigen targeting adapters and generation of bispecific CAR-T cells consisting of two or more different scFvs; however, the concerns of potential cytokine release syndrome, neurotoxicity, and B-cell aplasia remain.^144^

Another strategy for enhancing the antitumor efficacy of the CAR-T cells is to delete or express immunologic factors rather than CAR, named “Armored” CAR-T cells.^153,154^ Engineered CAR-T cells can secrete extracellular cytokines (including IL-12,^155^ IL-15,^156^ and IL-18^157^), antibodies, and bacterial enzymes increasing the prodrug concentration at the disease site.^158^ *C. perfringens* neuraminidase (CpNA) can remove sialic acid residues from the target cells. CpNA-restrained CAR-T cells exhibited superior effector function and cytotoxicity in vitro, showing enhanced antitumor efficacy in a Nalm-6 xenograft model of leukemia, xenograft model of glioblastoma, and syngeneic model of melanoma.^159^ Enabled CAR-T cells with additional natural or genetically modified transmembrane proteins can have more physiological functions such as signal transduction, recognition, and attachment. Kuhn et al. developed CAR-T cells to produce CD40L, a TNF-associated activation protein (TRAP), to recruit and activate immune effectors.^160^ Moreover, engineered CAR-T cells disturbing the intracellular transduction of inhibitory signaling pathways is another powerful strategy to inhibit the expression of immune-checkpoint molecules, including short-hairpin RNAs or CRISPR-Cas9.^161,162^

Although CAR-T therapy offers spectacular therapeutic opportunities against cancer, it has certain limitations such as tumor antigen escape relapse lowering the therapeutic efficacy. To conquer such challenges, novel strategies to optimize the CAR-T cells are needed for increased safety.

Certainly, the developments in SB will continue to promote the progress of tumor treatments; designing subtle gene circuits and finding novel biological components and the right targets could be the main endeavors. Meanwhile, controlling biosafety and reducing in vivo toxicity will also be necessary to balance cost and effectiveness.

## Engineered cell-based therapeutics for infectious diseases

Infectious diseases are the major reasons for death and disability worldwide.^163^ Pathogenic infections including those from bacteria, fungi, and viruses are treated by conventional antibiotic therapy, however, many antibiotics have side effects causing intestinal flora disorder, hearing impairment, nephrotoxicity, and allergic reactions. Moreover, the emergence of multidrug-resistant (MDR) bacteria urgently demands new treatment strategies.^164,165^ The developing SB could also provide efficient, accurate, and cost-effective tools to treat infectious diseases. Here, we mainly discuss novel strategies against infectious diseases from the SB perspective.

### SB tools against bacteria

Bacteria evolved with a variety of molecular weapons to kill or interfere with competitors, such as bacteriocins, effector proteins, and other chemicals.^166–168^ Thus, engineered bacteria can be exploited for novel antimicrobial biotherapeutics. Jana et al. introduced the arabinose-induced Type VI Secretion System (T6SS) into *Vibrio natriegens*, a safe bacterium, to manipulate the effector against competitors.^169^ Ting et al. developed programmed inhibitor cells (PICs), a new system expressing surface nanobodies to mediate antigen-specific cell-cell adhesion, exerting T6SS antimicrobial activity directly against selected bacteria.^170^ Bacteria use chemical language, termed quorum sensing (QS), to communicate in the community and regulate cell density.^168^ Probiotic *E. coli* designed to produce AI-2, which is naturally produced by *Vibrio cholera*, caused significant growth inhibition of *V. cholerae* and increased the survival rate of infected animals.^171^ Engineered Δ*alr* Δ*dadX E. coli* Nissle 1917 strain can produce Dispersin B, Pyocin S5, Lysin E7, and sense autoinducer 3OC_12_HSL secreted by *P. aeruginosa*.^172^ In the presence of 3OC_12_HSL, this *E. coli* strain triggers E7 lysozyme-mediated self-lysis releasing Pyocin S5 and Dispersin B that kills *P. aeruginosa*. In another case, attenuated human lung pathogen *Mycoplasma pneumonia* was reformed to secrete anti-biofilm microbial enzymes that degraded *Staphylococcus aureus* biofilm both in in vitro *and* in vivo.^173^ Lysostaphin-producing *Staphylococcus simulans* could treat methicillin-susceptible and methicillin-resistant *Staphylococcus aureus* infections.^174^ Although bacteria can be easily tailored to combat microbial pathogens, their use is limited by the on/off switch of effector release, potential toxic effects in human cells, and long-term stability.^175^ A new research direction has evolved combining Computer-Based Frameworks and SB for the discover of potential antimicrobial peptide (AMP).^176^

Producing selective endolysins (lysins) to break bacterial cells, bacteriophages are one of the most effective therapeutic approaches against both Gram-positive and Gram-negative bacteria.^177,178^ Furthermore, engineered bacteriophage may overcome the limitations of frequent escape of bacterial hosts by modifying cell-wall related receptors, CRISPR-Cas disruption, or restriction-modification systems. *E. coli* bacteriophage T7 encoding depolymerase Dispersin B (T7DspB) could specifically hydrolyze polymeric b-1,6-N-acetyl-D-glucosamine (PNAG) of bacterial biofilms reducing biofilm formation of *E. coli*.^179^ Masuda et al. generated recombinant lnqQ-T7 phage by integrating lnqQ (structure gene of Leaderless bacteriocins, LLB, lacticin Q) into the T7 phage genome, which significantly inhibited lacticin Q-susceptible *Bacillus coagulans* and *E. coli*.^180^ Hsu et al. engineered λ phage to express transcriptional repressor of Shiga toxin (Stx), which successfully reduced Shiga toxin production in the *E. coli* population colonizing the intestinal tract model.^181^ The phages can also effectively transport molecules (such as proteins, peptides, DNA, RNA, and drugs) to targeted bacteria. *Staphylococcus aureus* phage encoding an antimicrobial small acid-soluble spore protein (SASP) was developed to kill *S. aureus*.^182^ This engineered phage (SASPject PT1.2) showed 100% activity against the 225 geographically diverse *S. aureus* isolates. However, bacteriophage treatment will inevitably lead to bacteriophage resistance and serious side effects such as the release of bacterial endotoxins. Also, bacteriophage is highly specific and attacks limited bacteria. In future, SB and genome engineering can expand the scope of phage therapy.^182^

### SB tools against viral infection

SB-based tools can also contribute to the quick diagnosis, therapy, and prevention of viral infections.^26^ Wei et al. engineered *Lactobacillus jensenii*, a commensal bacterium that colonizes the cervicovaginal mucosa, to prevent HIV transmission; the engineered strain secretes two-domain CD4 (2D CD4) proteins,^183^ which associate with HIV type 1 (HIV-1) gp120 and inhibit HIV-1 invasion. Similarly, the probiotic bacterium *E. coli* Nissle 1917 was designed to secret HIV-gp41-hemolysin peptides, which blocks HIV fusion and subsequent invasion.^184^ This engineered strain could continuously and stably colonize the colon and cecum and released anti-HIV peptides for several months in mice. Furthermore, a genetically modified *Lactobacillus acidophilus* ATCC 4356 expressing human CD4 receptors on its surface can adsorb HIV-1 particles.^185^ The results showed that CD4-carrying bacteria could capture HIV-1 particles and attenuate infection efficiency in vitro. Martins et al. developed a potential microfluidic attenuator containing engineered *E. coli* to trap Ebola viruses in human blood.^186^ Along these lines, incorporating engineered probiotics into the vaginal microbiota can inhibit the spread of viruses.

Recently, COVID-19 (SARS-CoV-2) pandemic became a serious concern for public health safety,^187,188^ urgently requiring an efficient vaccine. Chen et al. engineered yeast to stably express the SARS-CoV-2 receptor binding domain (RBD) without impacting protein structure and functionality, which can be a promising candidate vaccine for COVID-19.^189^ Furthermore, the yeast expressed RBD combined with the 3M-052-alum provoked powerful immune response against SARS-CoV-2 in rhesus monkeys.^190^ Apart from recombinant proteins, nucleic acid-based (such as messenger RNA) vaccines are potential candidates against SARS-CoV-2. mRNA vaccines can transform host cells into “protein factories”, providing in situ the protein for a balanced immune response for both cellular and humoral immunity. In 2021, clinical trials demonstrated that 2 doses of mRNA vaccine BNT162b2 successfully induced a persistent germinal center B-cell response and robust humoral immunity. This made BNT162b2 to be the first licensed mRNA vaccine against COVID-19 by FDA for people over 16 years of age. Although a considerable number of mRNA vaccines have recently been developed, including PTX-COVID-19-B, SYS6006, and mRNA-LNP, they have several limitations including allergic reactions, uncertain immunity length, and difficult storage.^191^ Notably, SB tools also can help predict and identify the emerging SARS-CoV-2 variants. Based on reverse genetics, Thao et al. developed a rapid and robust yeast-based synthetic genomics platform for the genetic reconstruction of SARS-CoV-2, which can help generate and functionally characterize evolving viral variants in real-time.^192^ Scientists have used yeast screening to identify antibody escape mutants of SARS-CoV-2 S RBD to provide new therapeutic strategies against SARS-CoV-2. Importantly, viruses are evolutionarily autonomous; the emergence of new mutations led to the rapid global evolution of influenza viruses.^193,194^ Accordingly, the virus evolutionary genetic algorithm should be applied for the development of next-generation SB tools.

## Engineered cell-based therapeutics for imbalanced flora-related diseases

**Fig. 5:**
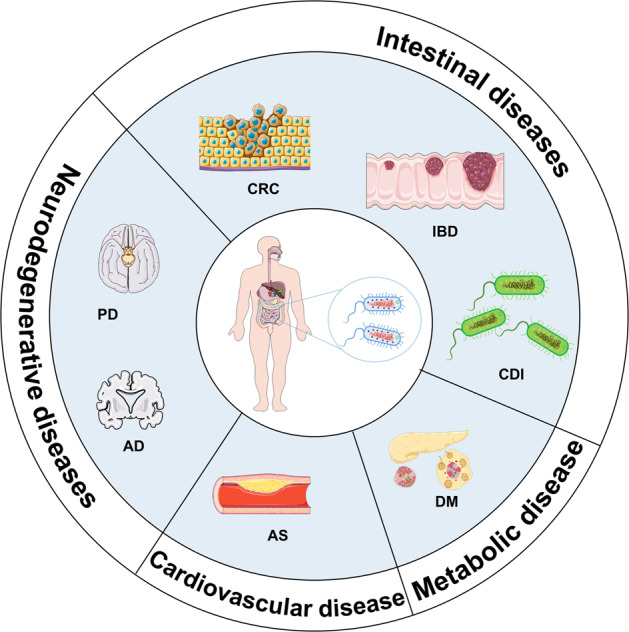
Microbial modulation for disease treatment. AD Alzheimer’s disease, PD Parkinson’s disease, CRC colorectal cancer, DM diabetes mellitus, CDI *Clostridium difficile* infection, IBD inflammatory bowel disease, AS atherosclerosis

The human body is inhabited by a multitude of microbial communities, which co-evolve with human hosts and are essential for their health.^195–197^ It is now well-established that human microbiome disturbance can lead to many chronic diseases, such as colorectal cancer (CRC), autism, and diabetes mellitus (DM).^72,198–201^ Microbial population modulation is a new treatment strategy for intestinal and neurodegenerative diseases (Fig. 5).^105^

### Intestinal diseases

*Clostridium difficile* infection (CDI), which causes gut dysbiosis, is a major cause of morbidity and death worldwide.^202,203^ Supplementation with Firmicutes, which convert primary bile acids to secondary bile acids, can significantly lower the growth and virulence of *C. difficile* by competitively colonizing the infected gut.^204^ Microbiome product Seres SER-262, which was isolated from healthy donors, could eradicate *C. difficile* infection. Altered composition, function, and metabolites of the intestinal flora have been associated with the progression of inflammatory bowel disease (IBD).^205^ Traditional IBD treatment is limited by side effects and complications. Lee et al. developed a hyaluronic acid-bilirubin nanodrug (HABN) which after accumulation restored the epithelial barrier alleviating inflamed colonic epithelia by restoring gut microbiota balance.^206^ Whelan et al. engineered the probiotic bacterium *E.coli* Nissle 1917 for the treatment of IBD, which secretes nematode immunomodulator cystine in the intestine.^207^ This modified probiotic significantly inhibited gut inflammation in murine acute colitis and can potentially treat other similar diseases. A *Lactobacillus* probiotic was engineered to produce anti-inflammatory molecules, in particular IL-10, in response to murine colitis.^208^ An engineered *E. coli*, named EcNL4, improved intestinal microbiota and thereby colitis in mice through the sustainable release of (R)-3-hydroxybutyric acid (3HB).^209^ An *E. coli* strain, named SYNB1618, expressing the phenylalanine ammonia-lyase and L-amino acid deaminase genes was engineered to consume phenylalanine in the gut. Overall, the above studies indicated that engineered bacteria can be potentially used to treat rare metabolic diseases.

### Neurodegenerative and other diseases

Neurodegenerative diseases such as Parkinson’s disease (PD), Alzheimer’s disease (AD), and amyotrophic lateral sclerosis (ALS) have similar features.^210^ Emerging evidence closely associates gut dysbiosis with the development of neurodegenerative diseases through the gut–microbiota–brain axis.^211,212^ Wu et al. showed that *Enterobacteria* infection strongly exacerbated neurodegeneration by promoting the recruitment of immune hemocytes to the brain.^213^ Being functionally related to the brain immune system, the intestine has been termed the “second brain”. A study showed that oral administration of engineered MG136-pMG36e-GLP-1 strain derived from *Lactococcus lactis* MG1363 continuously produced Glucagon-like peptide-1 (GLP-1), which dramatically reduced lipopolysaccharide (LPS)-induced memory impairment and 1-methyl-4-phenyl-1,2,3,6-tetrahydropyridine (MPTP)-induced motor dysfunction, and the abundance of Enterococcus and Proteus pathogens in mice.^214,215^ This strain could be a novel intervention in AD and PD.^214–221^ Notably, probiotics can compete with pathogenic bacteria for nutrients and binding sites and secrete/produce bacterio-toxins which repress the invasion and adhesion of pathogens.^218^ Oral bacteriotherapy with SLAB51 probiotic and p62 (SQSTM1)-engineered lactic acid bacteria inhibited the progression of AD, indicating the positive effect of the probiotic intervention in neuronal diseases.^219^ So far, *Lactobacillus* and *Bifidobacterium* strains are the most promising candidates for improving cognitive functioning by reducing the levels of oxidative and inflammatory biomarkers in AD.^220^

Engineered commensal bacteria also have significant potential in other chronic diseases, i.e., DM and Atherosclerosis (AS). Duan et al. engineered *Lactobacillus gasseri* ATCC 33323 to secrete GLP-1 (1–37) to stimulate the conversion of intestinal epithelial cells into insulin-secreting cells in a diabetic rat model. They showed that rats fed daily with engineered bacteria developed insulin-producing cells in the upper intestine, sufficient enough to ameliorate hyperglycemia.^214^ A recent study showed that an engineered strain MG1363-pMG36e-GLP-1 (M-GLP-1) dramatically decreased body weight and improved glucose intolerance in high-fat diet (HFD) obese mice.^215^ May-Zhang et al. developed a novel *E. coli* Nissle 1917 strain expressing N-acyl phosphatidylethanolamines, which are precursors of endogenous lipid satiety factor N-acylethanolamides; the strain significantly reduced the levels of adiposity, hepatic triglycerides, and fatty acid synthesis lowering serum cholesterol levels and extent of necrosis in atherosclerotic mice.^217^

Similar to chemical drugs or cell-based therapies, the safety, tolerability, and efficacy of engineered therapeutic microbes for chronic disease therapies have attracted widespread attention.^221^ The long-term persistence, potential sensitization, and influence of engineered probiotics on native gut microbiota are yet to be completely comprehended. With the advances in SB tools and massive clinical trial data, this field is anticipated to develop therapies for many chronic diseases in the future.

## Synthetic cells in drug discovery and development

**Fig. 6:**
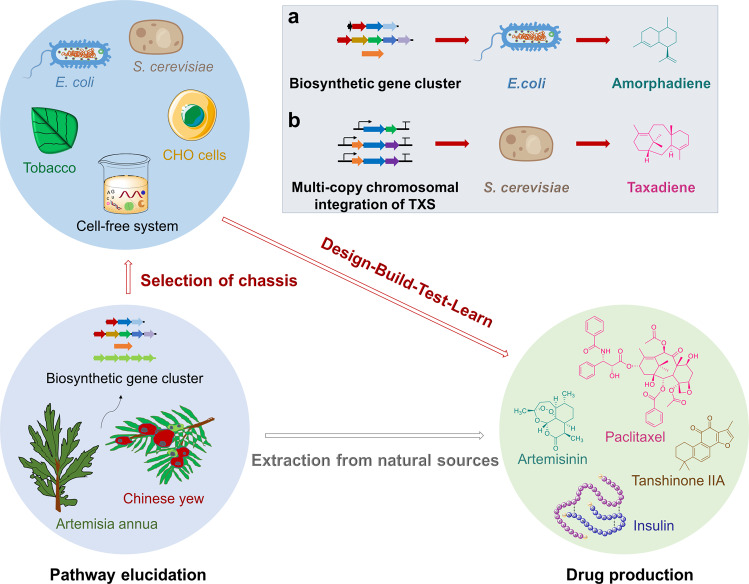
Synthetic biology facilitates drug production in diverse chassis systems. Outlined scheme of the typical pipeline of drug development consisting of pathway elucidation, chassis selection and drug production. Gray arrow indicates extraction of bioactive compounds from natural sources, red arrows indicate general synthetic biology pathways. **a** Synthetic biology production of amorphadiene in *E. coli* chassis. **b** Synthetic biology production of taxadiene in *S. cerevisiae* chassis

Microorganisms, plants, marine resources, insects, and even mammals have always been important sources of pharmaceuticals/natural compounds. In nature, such compounds exist in low concentrations and are often difficult to synthesize due to their structural complexity. Advances in SB allow the production of bioactive compounds in non-natural hosts, which has revolutionized the pharmaceutical industry. Using computational and experimental tools, SB can help design chassis cells as novel drug screening platforms or as biofactories for the production of difficult bioactive compounds (Fig. 6).

### SB in drug discovery

*E. coli* is the best-studied prokaryotic model organism with properties of a well-defined genetic background, rapid reproduction, low cost, and high levels of foreign protein expression. Therefore, it serves as an important component of high-throughput drug screening platforms. For example, multidrug-resistant *E. coli* strains are utilized to identify novel antimicrobial compounds.^222^ Male et al. developed a high-throughput gene coding screening platform to screen a SICLOPPS library of cyclic hexapeptides in *E. coli* that could inhibit the protein interaction between the human CMG2 receptor and the *Bacillus anthracis* protective antigen (PA).^223^ Van der Donk and co-workers generated a bicyclic peptide library in *E. coli* by combining the generation of a lanthipeptide library with a bacterial reverse two-hybrid peptide library (RTHS), and identified interaction inhibitors for the HIV p6 protein and UEV domain of the human TSG101 protein.^224^

*E. coli* lack the regulation mechanisms of eukaryotic gene expression and therefore is not suitable for screening bioactive compounds against eukaryotic targets.^225^ *Saccharomyces cerevisiae* is a commonly used eukaryotic model organism for the expression of active eukaryotic proteins undergoing post-translational modifications, including disulfide bond formation and glycosylation. In addition, *S. cerevisiae* can also tolerate a wide range of pH and high osmotic pressure, which makes it a robust organism in practice.^226^ *S. cerevisiae* strains, such as a yeast strain expressing human PARP1, have been used to screen inhibitors of enzymes involved in cancer progression.^227^ A yeast-based chemogenomic platform was used to identify the interaction between the chemotherapeutic agent methotrexate and the protein Dfr1 using haploinsufficiency profiling.^228^

The complexity of mammalian cells limits their engineering as host cells. However, recent developments in SB are changing the situation. For example, CRISPR-Cas9 technology was applied to create a high-throughput platform of primary human CD4^+^ T cells for the functional analysis of host factors in HIV infection and pathogenesis.^229^ Mammalian cells, such as Chinese hamster ovary (CHO) cell lines, are used for primary screening and production of complicated biopharmaceuticals. We will now discuss gene circuits based on mammalian cells. The mammalian cells have a high cost of production and also suffer from the risk of contamination from human-specific pathogens.

Synthetic gene circuits offer a huge opportunity for advanced drug discovery. Antimicrobial resistance (AMR) is emerging as a serious health threat, which can possibly be addressed by synthetic biology. Synthetic mammalian gene circuits can be used for the screening of novel antimicrobial compounds. Streptogramin-inducible (PipON) and streptogramin-repressible (PipOFF) mammalian gene-regulation systems were constructed based on the pristmamycin-responsive interaction between pristinamycin-induced protein (Pip) and its ptr promoter (Pptr) target sequence.^230^ The *Streptomyces coelicolor* Pip was adapted to modulate reporter gene expression (SEAP, secreted alkaline) in Chinese Hamster Ovary (CHO) cells in response to streptogramin antibiotics. This screening allowed the identification of new bioavailable and more sensitive antibiotics.^231^ Another example is the discovery of new anti-tuberculosis drugs. Ethylthionamide repressor (EthR)-based genetic circuit screening was used to identify 2-phenylethyl-butyrate, an anti-tuberculosis drug candidate against multidrug-resistant *M. tuberculosis*.^232,233^

### SB in natural drug production

Natural products are a valuable source of pharmaceuticals; however, they are difficult to synthesize and exist in low amounts in natural hosts. Rapid advances in SB now allow the production of high-value natural medicines and precursor compounds (Table 4).

**Table 4:** Advances in the research of high-value natural drugs and precursor compounds

Target drugs	Host	Products	Achievements
Artemisinin	*E. coli*	Amorphadiene	First production of artemisinin precursors in a microbial system^239^
	*S. cerevisiae*	Artemisinic acid	The synthesis of artemisinic acid in *S. cerevisiae* was achieved^240^
	*N. tabacum* and *P. patens*	Artemisinin	Production of artemisinin in model plants^242,243^
Paclitaxel	*E. coli*	Taxadiene	The first report of taxadiene production in *E. coli*^250^
	*E. coli*	Taxadiene	Taxadiene accumulation to 1 g/L.^251^
	*S. cerevisiae*	Taxadiene	Taxadiene accumulation to 129 ± 15 mg/L in *S. cerevisiae*^252^
	*E. coli* and *S. cerevisiae*	Oxygenated taxanes	Combining the strengths of *E. coli* and *S. cerevisiae* produced 33 mg/L oxygenated taxanes^254^
	*N. benthamiana*	Taxadiene and taxadiene-5α-ol	The first heterologous synthesis of taxadiene-5α-ol in a plant chassis^255^
Tanshinones	*S. cerevisiae*	Miltiradiene	A high yield of miltiradiene (365 mg/L) was obtained in yeast^257^
	*S. cerevisiae*	Miltiradiene	A high yield of miltiradiene (3.5 g/L) was obtained in bioreactor.^258^
	Hairy roots	Tanshinone	Enhancement of tanshinone content and antioxidant activity by hairy root metabolic engineering for the first time^259^
Opioids	*S. cerevisiae*	Thebaine and hydrocodone	Total biosynthesis of opioids in yeast^260^
Cannabinoids	*S. cerevisiae*	Cannabidiolic acid etc.	Total biosynthesis of cannabinoids in yeast^261^
Insulin	*E. coli*	Insulin A and B chains	Insulin was first produced in *E. coli*^263^
	*S. cerevisiae*	Proinsulin	*S. cerevisiae*-based expression system is efficient and secret human insulin^265^
Recombinant protein vaccines	*E. coli*	Trumenba^®^	Recombinant protein vaccine involving *E. coli* expression system^270^
	*S. cerevisiae*	Gardasil	Recombinant protein vaccine involving yeasts expression system^271^
	CHO	ZF2001	Recombinant protein vaccine involving mammalians cell expression system^272^
	*Sf9* insect cells	Recombinant COVID-19 vaccine	Recombinant protein vaccine involving Insect cells expression system^273^
AMPs	*E. coli*	GKY20	High expression level in *E. coli* by inclusion body formation^275^
	*E. coli*	Melittin etc.	Expression in *E. coli* using CaM as a carrier protein^276^
	*P. pastoris*	Apidaecin	Super heterologous expression and secretion of apidaecin in yeast^278^
Valinomycin	Cell-free system	Valinomycin	The first NRP that was fully biosynthesized in vitro using an cell-free system^282^
Nisin	Cell-free system	Nisin	Cell-free biosynthesis of nisin was achieved by a CFPS platform^284^

Semi-synthetic artemisinin is a successful case of SB-based synthesis. Artemisinin, a highly effective antimalarial drug, is a sesquiterpene lactone, which is naturally obtained from Artemisia annua (Asteraceae).^234^ Artemisinin and its derivatives also have anticancer,^235^ anti-inflammation,^236^ antiviral,^237^ and anti-SARS-CoV-2 activity.^238^ Initially, Martin et al. attempted the production of artemisinin precursors in microbial systems; a mevalonate pathway from *S. cerevisiae* and a synthetic amorpha-4,11-diene synthase (ADS) were heterologously expressed in *E. coli*, producing amorphadiene (Fig. 6a).^239^ Synthesis of artemisinic acid in *S. cerevisiae* was achieved using an engineered mevalonate pathway, amorphadiene synthase, and a novel CYP450 from the eukaryotic origin, all of which could not be perfectly expressed in *E. coli*; the synthesized artemisinic acid was transported out of the engineered yeast simplifying purification.^240^ A complete biosynthetic pathway, including a plant dehydrogenase and a second cytochrome, allowed the production of artemisinic acid in engineered *S. cerevisiae* at a fermentation titer of 25 g/L; singlet oxygen was used to convert artemisinic acid to artemisinin.^241^ Artemisinin was also produced in two model plants, tobacco (*Nicotiana benthamiana*) and moss (*Physcomitrella patens*).^242,243^

Paclitaxel (taxol), a diterpenoid secondary metabolite derived from the stem bark of Chinese yew (*Taxus brevifolia*),^244^ is widely used to treat breast,^245^ ovarian,^246^ pancreatic,^247^ lung,^248^ and prostate cancers.^249^ *T. brevifolia* resources are scarce and have paclitaxel in very low amounts. The chemical synthesis of paclitaxel is complex and costly. Therefore, the industrialized production of paclitaxel by attempted using the tools of SB. Taxadiene, an important intermediate in the paclitaxel biosynthesis pathway, was obtained for the first time by overexpressing DXP synthase, IPP isomerase, GGPP synthase, and taxane synthase genes in *E. coli* at a titer close to 0.5 mg/L.^250^ Multiple module metabolic engineering (MMME) in *E. coli* was used to divide the paclitaxel biosynthetic pathway into an upstream module (containing dxs, idi, ispD, ispF genes) for IPP and DMAPP synthesis and a downstream module (containing GGPPS, TXS genes) for the production of GGPP and taxadiene. The conditions for the optimal balance of the two pathway modules were determined by a systematic multivariate search, resulting in a taxadiene yield of ~1 g/L, which is the highest yield reported so far.^251^ Since several CYP450s (membrane-bound proteins) are involved in the biosynthesis of paclitaxel, eukaryotic yeasts are more suitable for the heterologous production of paclitaxel and its intermediates. In an *S. cerevisiae* system, multi-copy chromosomal integration of taxadiene synthase (TXS) harboring fusion solubility tags improved taxadiene titers (Fig. 6b). The effects of the selected promoter, Mg^2+^ concentration, TXS truncation length, chromosomal gene copy, and cultivation temperature were evaluated; a maximum taxadiene titer of 129 ± 15 mg/L was achieved after strain optimization.^252^ Recently, a statistical definitive screening design approach combined with the state-of-the-art high-throughput micro-bioreactors resulted in a taxane titer of 229 mg/L.^253^ A co-cultivation method for the production of oxygenated taxanes was established in a synthetic consortium combining the strengths of *E. coli* and *S. cerevisiae*, which produced 33 mg/L of oxygenated taxanes.^254^ Taxadiene synthase, taxadiene-5α-hydroxylase, and CYP450 reductase were introduced in plant *N. benthamian* to achieve high-level production of taxadiene and taxadiene-5α-ol using a chloroplastic compartmentalized metabolic engineering strategy combined with enhanced isoprenoid precursors.^255^ Although many excellent attempts have been made, more efforts are needed to optimize the complete biosynthetic pathway of paclitaxel for its industrial production.

Tanshinones, including tanshinone I, tanshinone IIA, tanshinone IIB, dihydrotanshnone I, and cryptotanshinone, are bioactive diterpenoid compounds produced from the roots of Salvia miltiorrhiza Bunge (Danshen in Chinese), which have a wide range of pharmacological activities including antibacterial, antioxidant, anti-inflammatory, cardiovascular protective, and antineoplastic properties.^256^ Modularized pathway engineering strategies were applied to create SmCPS and SmKSL fusion proteins with adjacent active sites and fused BTS1 and ERG20 to produce a high yield of miltiradiene (365 mg/L) in *S. cerevisiae*.^257^ By increasing the carbon flux toward GGPP and optimizing the gene module of miltiradiene synthases, the production of miltiradiene was further improved in *S. cerevisiae*, reaching 3.5 g/L in a 5-L bioreactor.^258^ Hairy roots are also an attractive system for drug production. Three genes, HMGR, DXS, and GGPPS, involved in the biosynthesis pathway for tanshinone were introduced in *Salvia miltiorrhiza* hairy root for a high-yield production of tanshinone with higher antioxidant activity.^259^

There have been several efforts for the heterologous production of small-molecule natural drugs based on SB approaches. The full biosynthesis of the opioid compounds thebaine and hydrocodone starting from sugar was engineered in yeast.^260^ The complete biosynthesis of the major cannabinoids, cannabigerolic acid, Δ^9^-tetrahydrocannabinolic acid, cannabidiolic acid, Δ^9^-tetrahydrocannabivarinic acid, and cannabidivarinic acid, starting from the simple sugar galactose was accomplished in *S. cerevisiae*.^261^

### SB in recombinant protein and nucleic acid production

Protein drugs mainly include recombinant protein drugs, monoclonal antibodies, and recombinant protein vaccines. Compared with small-molecule drugs, protein drugs have high activity, strong specificity, low toxicity, clear biological function, and favorable clinical application.^262^ In this section, recent examples of recombinant protein and nucleic acid production will be presented; an overview of typical examples is presented in Table 4.

Recombinant human insulin was the first recombinant protein drug. It was produced by the expression of chemically synthesized cDNAs encoding for the insulin A and B chains separately in *E. coli* and the disulfide bonds were formed under optimal conditions to obtain bioactive insulin.^263^ A new and more efficient method for human insulin production was developed using a PCR-based cloning strategy that optimized human insulin expression in *E. coli* using the pET21b expression vector. It eliminated the use of affinity tags, tedious insulin renaturation, inclusion body recovery steps, and the expensive enzymatic cleavage of C-peptide.^264^ An insulin secretion expression system in *S. cerevisiae* has been developed, which expresses a cDNA encoding for a proinsulin-like molecule. Threonine^B30^ was deleted for the fusion of proinsulin molecule with the *S. cerevisiae* α-factor prepro-peptide. This was later replaced by human proinsulin C-peptide with a small C-peptide. The purified proinsulin is subsequently converted into human insulin by tryptic transpeptidation.^265^ Recombinant human insulin has also been successfully produced in transgenic animals and plants, such as mice,^266^ *Arabidopsis thaliana*,^267^ and cucumber.^268^

The production of monoclonal antibodies (mAbs) has taken tremendous attention in the pharmaceutical industry. A modular synthetic biology approach is proposed to rationally engineer *E. coli* according to three functional modules to facilitate high-titer production of immunoglobulin G (IgG).^269^ Bacteria, yeast, insect cells, and mammalian cells are used to express recombinant protein vaccines. Trumenba^®^, produced in *E. coli*, was developed by Pfizer and uses two variants of the meningococcal factor H-binding protein as antigens.^270^ Quadrivalent HPV vaccine, Gardasil, is the first commercially available FDA-licensed HPV vaccine; the *L1* gene of four human papillomaviruses is expressed in *S. cerevisiae* and is used with an aluminum adjuvant.^271^ Most current COVID-19 recombinant protein vaccines are expressed in mammalian cell culture-based expression systems. ZF2001, consisting of tandemly repeated RBD-dimers and aluminum adjuvants, and the RBD-sc-dimers of MERS-CoV and SARS-CoV-2 both were produced at a high yield in an industry-standard CHO cell system.^272^ The West China Hospital of Sichuan University has developed a recombinant COVID-19 vaccine using baculovirus as a vector; the RBD region of the SARS-CoV-2 spike protein receptor binding domain is expressed in *Sf9* insect cells, which showed effective immunogenicity in phase I and phase II clinical trials.^273^ According to WHO, there were 54 recombinant COVID-19 protein vaccine candidates in the clinical phase as of June 17, 2022.

Peptide natural products exhibit diverse biological activities, however, the toxicity, limited bioavailability, and difficult mass production hinder their development as therapeutic agents. Natural peptides can be biologically synthesized with the help of SB,^274^ including recombinant production in bacteria, fungi, and cell-free systems. One way to reduce host toxicity is to produce peptides in *E. coli* as inclusion bodies. The antimicrobial short peptide (AMP) GKY20 derived from the C-terminus of human thrombin was fused with the C-terminus of Onconase for very high expression as inclusion bodies.^275^ Fusion proteins also have reduced toxicity for the heterologous expression host. Calmodulin (CaM), a common calcium-sensitive protein in eukaryotes, consists of two independent target binding domains with malleable methionine-rich interaction surfaces that can accommodate many basic and hydrophobic residues.^276^ CaM can be used as a universal carrier protein to express various types of AMPs in *E. coli*, such as melittin, fowlicidin-1, tritrpticin, indolicidin, puroindoline A peptide, and human β-defensin 3 (HBD-3). This strategy effectively masks the toxic activity of many AMPs and protects them from degradation during expression and purification.^276^ Recently, *E. coli* MSI001, a lethal strain, was isolated from mice; the strain was used for screening bacteria-binding heptapeptides based on an integrative bioinformatics approach, phage display technology, and high-throughput sequencing.^277^ Yeast is another promising cell factory for the production of peptides. Apidaecins, a series of small and proline-rich antimicrobial peptides act as a defense system against many drug-resistant bacteria. Apidaecin expression in *Pichia pastoris* was improved after the selection of a mutant strain that limits the loss of the integrated plasmid after induction.^278^ Additionally, *P. pastoris* has been used to produce AMPs such as cecropin^279^ and defensins.^280^ Cell-free systems provide another solution for the production of natural peptides. Valinomycin is a non-ribosomal peptide with antibacterial, antiviral, and antitumor activities.^281^ A highly efficient cell-free platform was developed for the rapid, in vitro total biosynthesis of valinomycin with a yield close to 30 mg/L; the cell-free protein synthesis system (CFPS) was coupled with cell-free metabolic engineering system (CFME) by mixing two enzyme-enriched cell lysates to perform the two-stage biosynthesis.^282^ Other natural peptide products, RiPPs, were also synthesized in cell-free systems. Nisin, a lantipeptide has antibacterial activity and is widely used in the food industry. The cell-free biosynthesis of nisin was achieved by the complete reconstitution of its pathway in a CFPS. The same system can also be used for the genome mining of nisin analogs, screening of mutants, and guiding the overproduction of lanthipeptides in vivo.^283^ In addition, four different lasso peptides were synthesized using cell-free systems, including known examples such as burhizin, capistruin, and fusilassin and predicted lariat peptides such as ellulassin from *Thermobifida halotolerans*. This system can rapidly generate and characterize novel lasso peptide variants.^284^

Nucleic acid vaccines are based on either DNA or mRNA and are amenable to SB design. The target nucleic acid is inserted into human cells, which then produce copies of viral protein. Most of the nucleic acid vaccines against SARS-CoV-2 encode the virus’s spike protein.^285^ INO-4800, a DNA vaccine candidate targeting the SARS-CoV-2 spike antigen,^286^ is currently in phase 3 trials. BNT162b2 and mRNR-1273 are the approved mRNA vaccines, both of these LNP-formulated nucleoside-modified mRNA vaccines encode PS2 spike protein of SARS-CoV-2.^287^ There are 16 DNA-based and 37 RNA-based vaccine candidates of SARS-CoV-2 in the clinical phase, together accounting for about 32% of the total vaccine candidates.

With the advancement in SB, bacteria can also be used as active drugs. An engineered *E. coli* (EcNL4) was shown to improve gut microbiome and ameliorate colitis in mice.^209^ A combination therapy of low-dose anti-CD3 with a clinical-grade self-containing *Lactococcus lactis* (appropriate for human DM application, secreting human proinsulin and interleukin-10) cured 66% of mice with new-onset diabetes.^288^ A *Salmonella typhimurium* strain was engineered to secrete *Vibrio vulnificus* flagellin B (FlaB) in tumor tissues for cancer immunotherapy.^289^ Virus-based vaccines, such as polio and measles vaccines, are created utilizing an inactivated or weakened pathogen while maintaining the antigenic properties^285^ Recently, Beijing Sinovac’s inactivated SARS-CoV-2 vaccine has been approved.

## Conclusions and prospects

From bacterial-based biosensors and drug development to efficient treatments against pathogens, previously untreatable cancers, and other intractable human pathologies, SB has opened up various possibilities.^290^ For instance, there are currently seven cancer cell therapies through clinical trials that were approved by the FDA by July 2022.^291^ Despite these successes, SB-inspired cell engineering in medicine still faces many challenges, such as the lack of available sensors for essential biomarkers. Current microbial diagnostic and theragnostic approaches mostly rely on the existing sensors while numerous proteins with potential applications remain to be fully elucidated.^38^ Genome editing and manufacturing is a costly and lengthy process. Also, the therapeutic efficacy and technical difficulties limit their clinical applications.^292^ In addition, precise control of cellular output in engineered cells is very challenging, including the issues of bacteria diffusing to other tissues or being quickly eliminated by the host immune system. CAR-T-cell therapy is hampered by persistent infusion into patients and susceptibility to tumor antigen escape.^293^ Thus, additional efforts are required to integrate biological, chemical, and mechanical aspects for next-generation cell engineering.

Due to its dynamic multidisciplinary nature comprising different projects, approaches, and definitions, SB is evolving with advancements in biology, pharmacology, materials science, physics, and computational biology.^294^ The novel synthetic gene circuits combined with “on/off switches”, suicide gene systems, or CRISPR-Cas9 can create more precise control strategies for diagnostics and cell therapeutics. CRISPR-Cas9 significantly eases genome editing at targeted position(s).^162^ The iCasp9 kill switch has been licensed for more than 20 clinical trials including CAR-T cell and other type cell therapies and may help overcome limitations of currently approved CAR-T-cell therapies.^295^ In addition, non-biological factors have also been incorporated in SB approaches, such as temperature, light, rays, and nanomaterials. The integration of chemical biotechnology such as nanomaterial-coated bacteria provides tremendous promise for efficient transport, long-term storage, and spatial-temporal control in host.^26^ Gujrati et al. engineered bacteria to secrete melanin-containing OMVs with high photothermal conversion efficiency for photoacoustic imaging and photothermal therapy of 4T1 breast tumor.^296^ Moreover, the tools of computational biology such as machine learning can change the approach of SB by improving the search for biological components, optimizing design, and reforming/creating novel elements.

Taken together, recent advances in SB-related cellular diagnostics and therapeutics can significantly transform healthcare paradigms. Currently approved CAR-T therapies and upcoming clinical trials of other engineered cell therapies also inspire the rapid expansion of SB. SB-based biofactories can contribute to the screening and development of new and affordable medicines, including recombinant vaccines. In light of this, SB-inspired cell engineering will enter a new perspective and expand its application for various disease treatments.
